# Navigating Affordances for Learning in Clinical Workplaces: A Qualitative Study of General Practitioners’ Continued Professional Development

**DOI:** 10.1007/s12186-022-09295-7

**Published:** 2022-07-07

**Authors:** Linda Sturesson Stabel, Cormac McGrath, Erik Björck, Agnes Elmberger, Klara Bolander Laksov

**Affiliations:** 1 Department of Learning, Informatics, Management and Ethics, Karolinska Institutet, Stockholm, Sweden; 2 Department of Education, Stockholm University, Stockholm, Sweden; 3 Department of Clinical Genetics, Karolinska University Laboratory, Karolinska University Hospital, Stockholm, Sweden; 4 Department of Molecular Medicine and Surgery, Karolinska Institutet, Stockholm, Sweden

**Keywords:** CME, Continuing medical education, CPD, Continuous professional development, General practitioner, Lifelong learning, Medical doctors, Medical specialists, Physicians, Workplace learning

## Abstract

Medical specialists’ lifelong learning is essential for improving patients’ health. This study identifies affordances for learning general practitioners (GPs) engage in, and explores what influences engagement in those affordances. Eleven GPs were interviewed and the interview transcripts were analysed thematically. Stephen Billett’s theoretical framework of workplace participatory practices was used as an analytical lens to explore the topic. Challenging patient cases were identified as the main trigger for engagement in learning. Local, national and international colleagues from the same and other specialties, were found to be an important affordance for learning, as was written material such as websites, journals and recommendations. Other inputs for learning were conferences and courses. Workplace aspects that were essential for GPs to engage in learning related to: place and time to talk, relevance to work, opportunity for different roles, organisation of work and workload, and working climate. Importantly, the study identifies a need for a holistic approach to lifelong learning, including spontaneous and structured opportunities for interaction over time with colleagues, establishment of incentives and arenas for exchange linked to peer learning, and acknowledgement of the workplace as an important place for learning and sufficient time with patients. This study contributes with a deepened understanding of how GPs navigate existing affordances for learning both within and outside their workplaces.

## Introduction

Research shows that formalised continued professional development (CPD) activities such as courses and conferences increase knowledge, change attitudes and behaviours, and improve physicians’ skills (Allen et al., 2019; Cantillon & Jones, 1999; Cervero & Gaines, 2015). However, other studies suggest that even if physicians’ knowledge improves, changes in practice and patient outcome seldomly occur (Pype et al., 2014) and knowledge is rarely shared amongst colleagues in the workplace (Cervero & Gaines, 2015). Also, physicians may experience challenges integrating new ideas into practice (Allen et al., 2019; Casey et al., 2020) with CPD activities focusing on an individual level (Cervero & Gaines, 2015; Eppich et al., 2016; Mundet-Tuduri et al., 2017), a lack of compliance or motivation, and insufficient resources in the organisation (Price et al., 2010). The impact of formalised educational activities may also decrease over time (Casey et al., 2020). As research indicates mandatory CPD systems may lead to participants developing instrumental approaches, where gaining credits is seen as the primary goal (Altin et al., 2014; Eliasson and Lundqvist, 2019; Yam et al., 2020) it is important to better understand other contexts for CPD than courses. Further, research about physicians’ learning is often limited to exploring learning in formalised programmes. Still, such programmes only constitute a minor part of physicians’ lifelong learning as they also learn in the workplace (Cuyvers et al., 2016; Mertens et al., 2018; Teunissen and Dornan, 2008).

By exploring physicians’ lifelong learning in the workplace we hope in this study to add valuable knowledge for clinical managers and physicians alike to enhance opportunities for workplace learning systematically. While the nomenclature in the literature is not always consistent, and identifies physicians in a broad sense, specialized physicians and medical specialists, this study focusses specifically on general practitioners (GP’s).

### Workplace Learning

As research foremost have focussed on formalised programmes, we start with an exploration of physicians’ workplace learning in general. Workplace learning may be integrated into formalised CPD to enhance implementation (Mundet-Tuduri et al., 2017). However, in order for this to happen, more knowledge about how health care organisations may facilitate workplace learning for physicians is needed. Learning situations in the workplace can be difficult to define as they have different levels of formality and the intention of individuals to learn may vary (Cuyvers et al., 2016; Eraut, 2004). In contrast to learning in formal educational settings, workplace learning often takes place unconsciously and unintentionally. Studies exploring physicians’ and medical specialists’ workplace learning, both in hospital and primary care settings, usually focus on experiences and activities that trigger learning (Cuyvers et al., 2016), learning through interaction with peers, other health professionals or patients (Cuyvers et al., 2016; Mertens et al., 2018; Riera Claret et al., 2020), and how materials such as computers, medical records, and literature mediate learning (Fenwick, 2014; Mertens et al., 2018). Still, there remains a paucity of research addressing how health care organisations facilitate lifelong learning for specialist physicians who have passed the residency training (Berkhout et al., 2018; Sherman, 2018; Vinas et al., 2020) and thus we need to better understand how medical specialists develop their knowledge in the workplace. We chose to focus on GPs, the largest specialist group, in this study.

Stephen Billett’s theoretical framework on workplace participatory practices (WPP) is used in this study to explore GP’s lifelong learning. According to Billett (Billett, 2001), workplaces afford opportunities for learning, by inviting individuals to engage to different degrees, providing access to activities using different tools, aims and goals, and through the values, norms and procedures inherent in the environment. Such opportunities for learning are referred to as affordances. Individuals choose to engage in these affordances according to their personal history, agency, intentionality, preferences, norms and habits (Billett, 2001, 2016). Billett’s WPP theory thus differs from other sociocultural theories, such as Wenger’s communities of practice theory (Wenger, 1999), in that individuals’ contributions to learning opportunities are emphasised (Billett, 2008). WPP acknowledges that individuals’ agency and engagement is framed by the sociocultural environment, which presents both opportunities and obstacles. Hence, workplace learning, even though it is not formalised, can be highly structured and inherently pedagogical (Billett, 2001). WPP theory moves beyond the dichotomisation of formal and informal, and emphasizes a holistic approach to lifelong learning where different learning opportunities are afforded and learning is the outcome of the interdependence between the affordances and individuals’ engagement. As a consequence, we argue that health care organisations may need to take a holistic approach to lifelong learning. However, what such a holistic approach entails may not be easily captured.

In summary, to facilitate for medical specialists to keep up with the constant increase in knowledge research shows we need to go beyond formalised courses and explore the workplace as context for lifelong learning. With this knowledge managers can get a valuable knowledge basis for enhancing possibilities for learning (affordances) specialists may engage in. In this study we focus on GPs, as this group has been identified as a group of specialist physicians with particular challenges regarding CPD as they often work in isolation, are less engaged in research and teaching and work under time pressure which leads to inadequate self-assessment of learning needs (Cantillon, 2016). Therefore, the aims of this study are to identify the affordances for learning in which physicians engage, and explore what workplace aspects influence their engagement in those affordances.

## Method

This study adopted a qualitative approach to enable an in-depth exploration of GPs experiences and perspectives on their learning (Cleland, 2015). Further, we used Billett’s WPP framework as an interpretative lens. To strengthen the trustworthiness and credibility of our study, we explain the context, recruitment of participants, data collection, analysis, and the research group below. The study was designed from a social constructivist perspective, and acknowledge the value of dialogue and iterativity throughout the research process.

### Context and Recruitment of Participants

The study was conducted in Sweden, where medical specialists’ training is outcome-based and usually lasts five years, including residency and formal educational activities. In Sweden, general practice (or family medicine) is a medical specialty, and GPs usually work in primary health care together with allied health care professions at government funded health care centres. GPs who had finished their residency training more than 2 years ago, were interviewed for this study and they were selected using a non-probability sampling method, using both convenience and snowball sample (Etikan and Bala, 2017). Eleven interviews were conducted, nine females and two males. They received information about the study via email and gave their written consent before the interview.

### Data Collection and Analysis

An interview guide was developed based on Billett’s theoretical framework, and was modified after pilot interviews. The interview guide consisted of the themes: *professional background, workplace learning, participation in external formal activities,* and *knowledge transfer* (see Appendix). All eleven interviews (60–80 min in length) were done by LSS online in Zoom between February and June 2021, were audio-recorded and transcribed verbatim. Interviews were conducted until the interview data were assessed not to generate any new insights to our analysis, through an iterative process of interviewing, transcription, data familiarization and reading of transcripts. The assessment was based on an evaluation of how research aim, quality of interviews, theoretical framework, and the type of study were aligned (Varpio et al., 2017).

Billett’s framework about workplace affordances and engagement was used as a lens for interpretation and a thematical content analysis was applied (Braun & Clarke, 2006). All interviews were read through for familiarisation (LSS), initial ideas were noted, and codes created (LSS). In addition, authors AE, CMG, EB, KBL read and coded two transcripts each. Codes were sorted into themes and sub-themes (LSS), then discussed and rearranged to establish the relationship between the codes and the themes. The iterative process moved between focusing on each theme and sub-theme, and zooming out to identify patterns across all data.

All authors are researchers in medical education but with different disciplinary backgrounds and competences: medicine (AE, EB), ethnology (LSS), pedagogy (LSS), sociology (KBL), and philosophy (CMG). Two are medical doctors (AE, EB) of which one is a specialist (EB), and two are educational developers (KBL, CMG). The researchers’ different perspectives enabled us to see the data from different angles with repeated discussions on interpretations of data, thus contributing to the trustworthiness of the results. None of the researchers had any previous relationship to the interviewees.

## Results

**Fig. 1:**
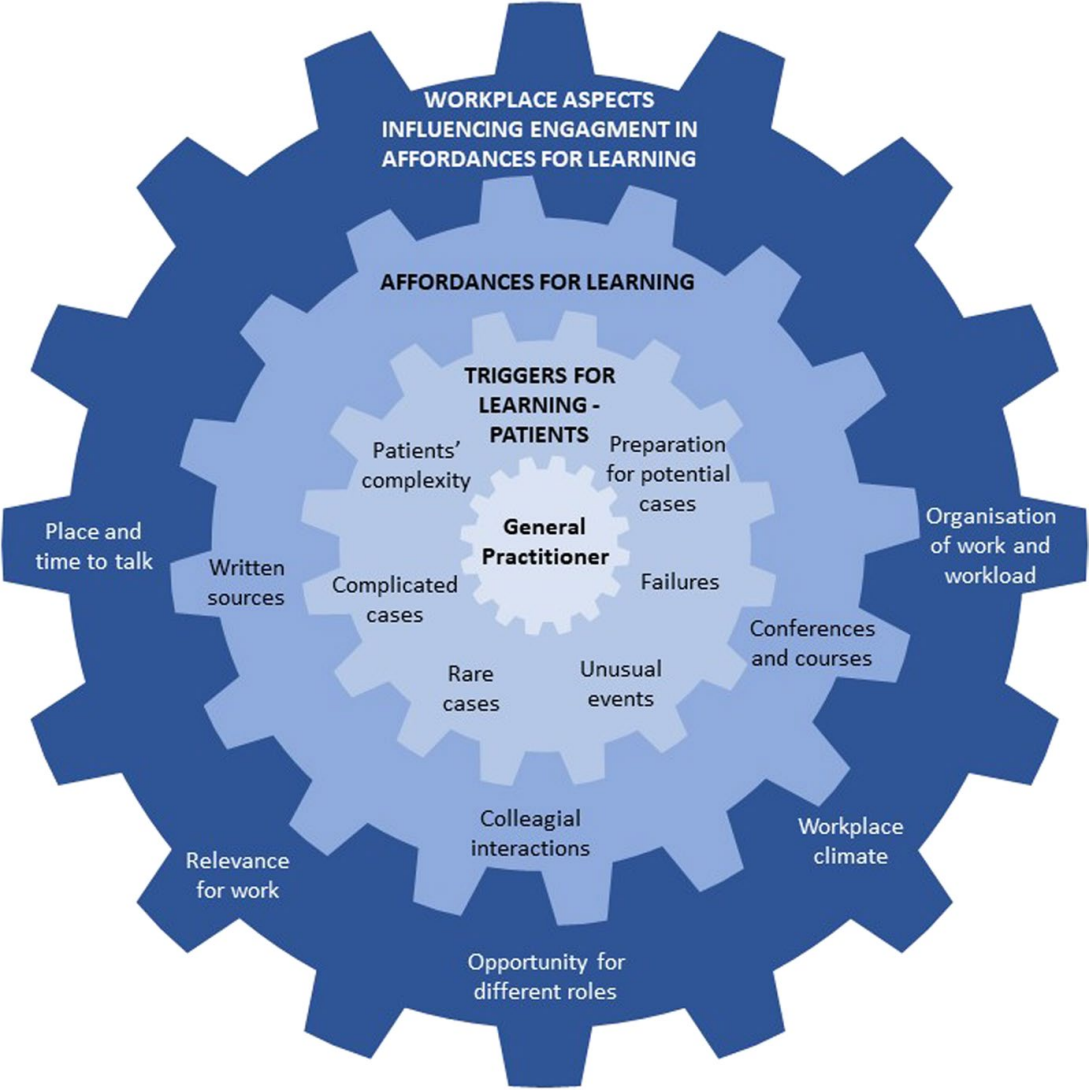
Themes and subthemes. Triggers for learning, affordances for learning, and workplace aspects influencing GP’s engagement in affordances for learning

The first result to be reported is *Triggers for Learning*, i.e., the different factors that triggered medical specialists’ learning. Secondly *Affordances for Learning* is presented, which reports on the different elements that afforded opportunities for learning in different settings. The third and final result is *Workplace aspects Influencing Engagement*, which relates to how the workplace enabled or presented obstacles to learning*.* Below, each theme is detailed with anonymised quotes from the interviews. The themes and sub-themes, and how they are connected as different dimensions are visualised in Fig. 1.

### Triggers for Learning: Patients

In this section we present the identified triggers for learning. Patients, and especially complicated patient cases, and the overarching goal of improving their health, were identified as the main triggers for learning. The interviewees reported that specific patient cases triggered their need to learn in specific instances but also played a role over a longer trajectory (e.g., when a patient case could not be resolved immediately).

The interviewees reported that the uniqueness of patients’ cases brought about challenges in the clinical setting, for example, when patients reacted differently to the same treatments, or had various coexisting diagnoses and drugs that interacted. Such challenges triggered a need to learn as these often complex and complicated patient cases were difficult to address without acquiring new knowledge. Rare patient cases were another trigger that led to GPs feeling a need to refresh their knowledge. Unusual events, such as the Covid-19 pandemic, also triggered and forced engagement in learning. Even if the interviewees had previous knowledge of similar phenomena to lean on, they still described a pressing need to learn.

Finally, failures such as treatment mistakes or conversations with dissatisfied patients were triggers for learning and changes in practice. For example, one GP suggested to a patient that they read up on a topic, but the patient found this advice unprofessional, which led to reflection and the GP changing their way of giving information to patients. Another GP once missed a serious diagnosis and had since then always had it in the back of her mind when meeting other patients with similar symptoms. The GPs’ awareness of potential future patient cases also led to a proactive engagement in affordances for learning (i.e., they had learned from experience that it was sometimes good to be prepared), as they never knew what patients may turn up. For more detail on what the interviewees said in relation to patients as triggers, see Table 1.

**Table 1:** Patient-related factors that drive engagement in affordances for learning and trigger continuous learning

Factor	Description and supporting quotations
Patients’ complexity	The fact that all humans are different, combined with general practice being a broad specialty. For example, patients may react differently to the same treatments and drugs, or have coexisting diagnoses and drugs that interact “Especially when it is complex stuff we work with, often, if you have a patient who has perhaps up to ten different conditions, and some are chronic and others acute, and they have ten different substances that might interact and influence each other. Then it is good to check, hey, would you do the same as me? Are we thinking the same way in this situation?” (GP 3)
Complicated patient cases	Patient cases difficult to proceed with and solve, and sometimes not solved at all. GPs usually thought back and forth and tried different tracks “If we have some unusually complicated cases where you have explored, made several checks and you don’t get anywhere, then you can ask: what track, what did I miss? How can I go forward? Then I may have those types of discussions.” (GP 10)
Rare patient cases	Patient cases that were rare and seldom occurring in the GPs own practice, urging a need for updating forgotten and/or new knowledge “There are things that are rare and then you forget about them, when it comes once a year or once every two or three years. Then I have to read, so I do.” (GP 8) “I had another, where no one understood what she had, and then there was one of those one-in-a-million in Sweden per year, and then there was somebody who had remembered those chapters in some course: ‘Ah, I wonder if it could be that strange condition’, and it was. Well, you are a team and you have to say it is needed.” (GP 1)
Unusual events	For example, the pandemic Covid-19 was new and, even if they had knowledge of similar phenomena to lean on, this was not enough “I don’t know how much time I have spent reading, but it has been a lot more this year when Corona came, that I can say […] But I read all the time. I do not sit and look at Facebook just to see what my friends are doing but I follow forums with scientific articles to keep up.” (GP 1) “There is a guy in Uppsala who has been absolutely fantastic at compiling articles about the Covid pandemic. Now it is about vaccines. I’m not active myself in writing back, but I read.” (GP 1) “There is a group [on social media] where you talk about Covid and you also get interesting studies that you can check out, to update yourself in Covid for example.” (GP 4)
Failures	Failures and mistakes made during practice, both one’s own and others “Once I suggested a patient to read more on a topic as a complement, but the patient found this advice unprofessional. Then, I thought later that one should ask more openly if they are interested in trying to read. I should try to first learn if they want to hear such advice.” (GP 2) Another GP mentioned a serious diagnosis that she missed, which was always in the back of her mind when meeting other patients with similar symptoms: “I missed a very serious pulmonary embolism disease because she [the patient] and I had no really good contact. I have never forgotten this because I was lucky that she didn’t die and I have never missed a pulmonary embolism again and I probably would not have experienced it so clearly if it had not been my fault in some way. […] You get a little scared, and I have heard that from other colleagues as well and it is almost everyone who has seemed to agree that they once made a wrong decision also got stuck, so you are much more aware of that diagnosis and take it into your, one usually says differential diagnostic thoughts. It can be this or this or this and then this diagnosis is always with me now when you talk breathing problems for example.” (GP 6)
Preparation for potential patient cases	Having potential patient cases in mind, thus preparing on what may be relevant in the future “Sometimes, that is why I read it [journal by the medical association] from cover to cover because you never know when that will come. And it’s also an aspect of this that makes me like general practice and the breadth of it all. I like to keep track of most things.” (GP 3)

Specific patient cases thus triggered GP’s learning and engagement in different affordances for learning.

### Affordances for Learning

When GPs were triggered to learn, they engaged with different affordances for learning, which are presented in this section under the theme of *written sources, conferences and courses*, and *collegial interactions*.

#### Written Sources, and Conferences and Courses

Written sources, both physical and digital, were either sought by the interviewees or shared with them. This material included medical journals, regional guidelines, recommendations from the National Board of Health and Welfare (a Swedish state agency working to ensure good health and high-quality care for the country’s inhabitants), as well as pamphlets about new treatments and medicines. These sources were useful to the GPs as they supported decision-making for patient care. Written sources served as affordances for learning either as a direct consequence of a patient encounter or through an indirect connection to patients where the relevance of the written source was highlighted:

“I scan the [journals] and see if there is anything that I think is relevant to my work. I read the articles a little more in depth, otherwise it is quite fast reading.” (GP 8)

The most frequently used written sources engaged in were specially adapted for GPs, concise, and had been approved by experts in the field:

“The literature is sorted on the websites and in the guidelines. I would not go back to an original article to try and find my own answer. You trust the experts’ opinions. I’m just glad that someone sorted the information for me – someone I trust. You cannot read all that yourself.” (GP 6)

Original research articles were seldom used because most patient cases were perceived as routine. However, further consultation of research literature could occur when cases were complicated. Written sources could lead to other forms of engagement such as interaction with colleagues.

Courses and conferences were also identified as contributing to the medical specialists’ continued professional development and continued lifelong learning. Not only did these events contribute to updating their medical knowledge, but also strengthened the GPs’ motivation:

“You become more inspired to work the day after, I’ve noticed. You feel a bit better – I have had time to devote to my own learning now. It’s a good feeling.” (GP 2)

The GPs valued the break created by participating in a course, as it gave time for reflection and a sense of doing something for oneself, as well as the opportunity to share experiences with colleagues from other settings and workplaces.

#### Collegial Interaction

Another important affordance for learning was the interaction with colleagues. Interaction with colleagues mostly took place in the workplace, but also in other contexts and with other specialties and professionals. Collegial interaction had four sub-components, namely *GPs at the workplace, physicians in residency, colleagues/peers in settings other than the workplace,* and *colleagues/peers in other medical specialties and professions.*

##### GPs at the Workplace

Colleagues at the workplace were approached about patient cases – usually after an independent search of online written sources. The urgency of a patient case determined the level of engagement; a more urgent case often led to immediate contact, while less urgent cases could be discussed at prearranged times. Colleagues were also approached for second opinions.

“You have a patient with these symptoms and you notice how your colleagues think and so on, and some are incredibly well-read and good at unusual things and then you can kind of free ride and learn things on the way.” (GP 5)

Reading peers’ notes in patient records also promoted learning. Several interviewees referred to: spontaneous discussions about patient cases that mediated learning, conversations during breaks, planned meetings focused on patient cases, presentations from colleagues who had visited conferences and courses, and lectures given by a colleague. Giving presentations to colleagues also led to learning as the interviewees had to update their knowledge. Many interviewees participated in weekly meetings where unsolved patient cases were discussed, and these meetings also triggered learning.

##### Colleagues/Peers in Residency

Students during vocational training, junior physicians doing their internships, and physicians in residency training provided the specialist with possibilities to learn and keep their knowledge updated:

“Every other week, [we have] structured in-house education where we educate each other. We let those who are doing their residency training, as part of their leadership training, educate all of us, so that we keep up to date with the latest regulations and guidelines on public health issues.” (GP 1)

Unstructured situations when the interviewees interacted with junior colleagues also led to learning, for example when participating in patient encounters as supervisors. The interviewees then had the opportunity to observe their juniors’ knowledge when interacting with patients, which in turn could develop interviewees’ knowledge.

##### GP Colleagues in Settings other than the Workplace

The interviewees sometimes contacted former co-workers or study peers to discuss patient cases, and this served as an affordance for learning. Socialising with GPs from other places during conferences and courses, or on social media forums consisting of thousands of GPs, were other examples of affordances. Different social media groups could either focus on specific subjects such as dermatology and the Covid-19 pandemic, or have a more general content. In these groups, information, questions, debate posts, and scientific articles were shared.

“We all review skin moles […] to select those to be removed and the good ones, and which ones should be sent to a dermatologist. […] it is good to practice. The more you see…and there is a Facebook group where somebody who is very skilled at this, each week posts cases and everybody can write what they think they see, and you get the correct answer a few days later.” (GP 3)

The interviewees stated that they seldom posted or commented in these groups, but read and sometimes used the information.

##### Colleagues in other Medical Specialties and Professions

GPs often consulted their colleagues/peers in other specialties on specific patient cases. When referring patients to specialists, the subsequent communication about the cases also contributed to the GPs’ learning, as did their reading the patient notes from the specialist after the referral. The GPs also contacted the authors of research papers if they needed to deepen their knowledge, but this was a rare occurrence.

“Last time I had a patient [with] lymphoedema […], I found an article in the *Läkartidningen* [medical journal published by The Swedish Medical Association] so I called the author and asked!” (GP 11)

Other specialists were also invited to give lectures on their expertise when it was relevant to the practice.

Cooperating or discussing patient cases with other health care professionals also led to learning:

“I also ask nurses questions. They are very good at wound care and leg ulcer treatment, which I know very little about and they sometimes have very good ideas.” (GP 6)

Collegial interaction led to continuous learning – often when colleagues helped to solve patient cases. Collegial discussions were sometimes about processing information and knowledge, wanting to know more about a topic or needing more detail, confirming or testing one’s knowledge, and reasoning about how to proceed with a patient case when there was no obviously right approach.

### Workplace Aspects Influencing Engagement in affordances for learning

In this section, we report on the workplace aspects that influenced engagement in the presented affordances. The following sub-themes are presented: *place and time to talk*, *organisation of work and workload*, *relevance for work*, *opportunities for different roles*, and *workplace climate*.

#### Place and Time to Talk

Where and when collegial interaction occurred was determined by the workplace culture and unwritten policies. Some workplaces had open-door policies that enabled engagement with colleagues. Other workplaces had policies limiting interruptions during patient appointments, which reduced the possibility of spontaneous conversations that potentially could facilitate learning. Several interviewees continually engaged in patient case discussions during lunches and breaks as this was considered an interesting topic. During lunches, they could discuss difficult patient cases, sometimes solving them just by talking to each other. Some workplaces had policies prohibiting discussions about patients in public spaces, citing concerns around patient confidentiality. Unwritten policies determined the possibility of initiating patient case discussions during lunches and other breaks.

“[Lunch break] is about half an hour, if that, and sometimes you are in a situation where there is a lot of ‘patient talk’ […] but there is some kind of policy that you shouldn’t because you need your time off.” (GP 9)

Interaction with colleagues was enabled mostly by setting aside time for weekly meetings, which were dedicated to patient case discussions, but also provided an opportunity to report on conference or course participation. These meetings were scheduled outside patient hours and participation in them was more or less mandatory.

“You have to be forced to discuss patient cases with colleagues [at meetings] or it will always be less prioritised than patient appointments. You have to schedule and say ‘Now it is time for education and everybody needs to join.’” (GP 6)

Meetings including patient case discussions were perceived by the GPs as important for many reasons, but primarily because they provided access to the knowledge, skills, judgement, and experience of several other colleagues. Also, the devoted space to meet and discuss was perceived as meaningful since the GPs otherwise worked alone. The lack of a structured space for learning and knowledge development had in some cases led GPs to transform their lunch break into a weekly learning activity since the organisation did not offer this needed space elsewhere.

#### Organisation of Work and Workload

Heavy workload, stress, and a lack of time hindered the GPs’ from having collegial discussions, reading written sources, and participating in conferences and courses. They usually had a number of patients for whom only they were responsible and they usually did not share patients or cover for each other if someone attended a conference. The interviewees reported that being a GP meant building long-term professional relationships with their patients. For many GPs, their personal interest in patients was a driving force for learning, but ironically their engagement in learning risked being suppressed by a heavy workload (i.e., a large number of patients). The endless flow of patients and a workload that would pile up if attending a conference decreased the GPs’ motivation to participate:

“The obstacle is that if I am away one day, yes, partly if it comes a little unplanned, then you have to re-book these patients, but then […] … my patients often still want to come to me so you still have the job.” (GP 9)

Financial constraints and bureaucratic procedures were also identified as obstacles to learning:

“Stockholm’s compensation system for care units is based a lot on how many patient contacts you have in a day and lots of things like that … so even if you were to take away administrative time, I do not think that it would be filled with: ‘Oh, how good, now we have four hours of discussion time’, but I think it would be filled with: ‘How good, now you can take an extra four hours’ worth of patients.’” (GP 3)

The possibility of participating in conferences also depended on when one had last attended a conference and on colleagues’ desire to also take part in the same conference; obviously, not everyone could join the same conference as it would halt the daily work. However, solutions for this were possible:

“They overlap, the programme is doubled, so half [of us] join the first time and the other half join the second time so you don’t have to shut down practice during one day. That wouldn’t work.” (GP 8)

Heavy workload, stress, and lack of time indirectly forced patient case discussions as the GPs did not have time to leave the workplace for lunch, which resulted in spontaneous discussions about patients.

#### Relevance for Work

The GPs were more likely to engage in affordances for learning that were relevant to their patients’ needs and circumstances (e.g., patients’ socioeconomic background or specific illnesses or conditions). The workplace context thus guided the direction of their continuous learning, albeit with the manager having the final say on which courses and conferences GPs could participate in. For example:

“It cannot be the same people who [always] participate, and someone who wants to learn everything about nail fungus year in and year out. It is still the manager who decides: yes, but this is what you need to know to practice.” (GP 1)

The notion of relevance and meaningfulness also required that colleagues presenting their experience of a course or conference cherry-picked only the most relevant parts.

#### Opportunities for Different Roles

Workplace conditions also allowed the GPs to take on other roles that afforded learning opportunities (e.g., being a formal supervisor for junior colleagues or giving lectures in the workplace):

“When they [students] ask, you are sometimes forced to read up on things that you don’t really know about or have forgotten. So, it’s always good. And there is a lot of discussion about patient cases.” (GP 2)

Some GPs saw themselves as resources for learning, trying to facilitate younger colleagues’ learning and knowledge development by taking on an informal role:

“For educational purposes, you want to ask this question: what do you think, this patient is so-and-so years old, and has these symptoms, what is your first thought?’ […]. We do this and it’s really interesting.” (GP 7)

In some workplaces, they had introduced ‘in-house resource physicians’ whose job was to redirect queries from those having patient encounters. The GPs took turns acting as resource physicians and were sometimes asked to find answers to questions they would not normally be asked. However, if the resource physician was not expected to be knowledgeable about the specific case, they were usually not approached.

#### Workplace Climate

The workplace climate needed to be open, trustful and safe in order for colleagues to serve as affordances for learning. A safe environment enabled the interviewees to admit knowledge gaps, remain open to questions, but also ask questions and share mistakes for others to learn from. Some interviewees took on the responsibility for creating this safe workplace climate by asking questions to which they knew the answer, and sharing their own mistakes.

“You open up for it if you do it yourself. I mean, then it becomes less of a shame. If I with my double specialist education and my PhD say I did something and it was not good, then the threshold is lower for somebody else to say it too.” (GP 6)

Managers were seen by the GPs as important in creating a good working environment by, for example, participating in meetings and making sure that everyone had space to speak. Signs of a poor working environment included reports of high staff turnover and heavy workload.

In summary, patient cases (especially complex, complicated, and rare ones, but also unusual events, failures made and preparation for future patient cases) triggered GP’s learning and their engagement in the different affordances for learning: written sources, conferences and courses, and colleagues. Colleagues as affordances for learning were located at the workplace and in other settings, and had various roles, medical specialities, and professions. GP’s engagement in affordances were further influenced by workplace aspects related to having place and time to talk, how the work was organised and workload, the affordance’s relevance for work, having opportunities for different roles, and workplace climate being open, trustful, and safe.

## Discussion

The aim of this study was to identify the affordances for learning that GPs engage in, and explore what workplace aspects influence such engagement using Billett’s workplace participatory practice framework as an analytical lens (Billett, 2001). Previous research suggests differences in drivers for formalised and workplace learning (Hilkenmeier et al., 2021). This study adds to this knowledge by identifying how GPs navigate affordances for learning in clinical workplaces and hence, offers a better understanding of how health care organisations may facilitate lifelong learning for GPs from a more holistic perspective. The discussion will now consider different elements of workplace learning (patients as triggers, interdependence between affordances and engagement, workplace conditions and individual engagement) and how they are intertwined in relation to GPs’ lifelong learning.

### Challenging Patient Cases as Triggers

Unsurprisingly, the findings of this study show that challenging patient cases were the primary trigger of GPs’ learning. The findings highlight the importance of triggers for learning, which is an aspect not specifically highlighted in Billett’s work (Billett, 2001). Patients triggered engagement in affordances for learning, both directly and indirectly. *Directly* in the sense that rare or complex patient cases meant that GPs sought affordances for learning immediately, and *indirectly* as learning was also mediated via less urgent patient cases, previous encounters, and preparation for future patients. The GPs’ focus was directed towards patients’ health and well-being, continuously processing and anchoring problem-solving and knowledge in practice. In line with the WPP framework, learning could thus be seen as a side effect of professional practice. Since patients are triggers for learning, it is thus crucial for the organisation to ensure opportunities for the establishment of strong relationships with patients, for example by offering the opportunity to meet the same patient over time.

### Interdependence between Affordances and Engagement

Billett’s WPP framework emphasises the interdependence between individuals and their workplace environment in generating learning (Billett, 2001, 2004). This study aligns with this view and clarifies the relationship. The interviewees described affordances for learning as invitational to different degrees. Written sources, for example, were particularly appreciated when short and concise and adapted for GPs. Access to formal activities was also emphasised as valuable when, for example, they were offered twice during one day enabling more GPs from the same clinic to participate. Consequently, engagement in affordances for learning was focused on authentic learning opportunities, instead of fulfilling demands of CPD credits (Altin et al., 2014; Yam et al., 2020).

Within general practice as well as in other specialties, discussions with peers are highly valued when learning (Cook et al., 2017, Cuyvers et al., 2016). Similarly in this study, colleagues in the workplace play a key role in GPs’ lifelong learning (both inter- and intra-professionally), as well as colleagues in other settings in constructing collectively shared guidelines for practice in what Gabbay & Le May have called ‘mindlines’ – collectively reinforced and internalised tacit guidelines (Gabbay & Le May, 2004). In addition, studies on GPs’ learning show that formal CPD activities located outside the workplace are preferred when including possibilities to discuss and share experiences from one’s own clinical practice (Dowling et al., 2020; Kjaer et al., 2015; Pype et al., 2014), and that these discussions also facilitate changes in practice (Dowling et al., 2020). An interesting finding in this sense was that, even though the GPs had immediate access to an unlimited amount of knowledge (e.g., online), colleagues still played an important role in the learning process, offering each other a sounding board to test ideas and a forum for listening to the experiences of others. Experienced colleagues mentoring newly qualified specialists have been suggested to play a similar role (Cuyvers et al., 2016). This study thus suggests that this pattern continues when practicing as a GP for several years, and that interaction with students/trainees also affords learning. In this way, these findings expand the WPP framework in that they identify a need for a holistic perspective on workplace structures that acknowledge affordances for GPs’ learning both within the current work setting but also beyond it, together with colleagues outside one’s own physical work setting.

### Workplace Aspects that Facilitate Lifelong Learning

Different aspects of the workplace hindered or facilitated engagement in affordances for learning. Unsurprisingly, a heavy workload and lack of time were identified as obstacles to learning. As for physicians in other specialties (Cook et al., 2017, Price et al., 2010; Yam et al., 2020), lack of time is one of the most commonly mentioned barriers to participation in formal learning activities for GPs, and it also hinders learning in workplaces (Mertens et al., 2018). From this study, the system with remuneration per patient seems to reinforce the sense of lack of time. For health care organisations to make the most of physician participation in learning activities the creation of a holistic approach that facilitates learning is key. This study suggests that when striving to develop lifelong learning in workplaces it is essential to pay attention to how patient workflow is organised between colleagues, what financial incentives from external stakeholders are present, and how long-term relationships between GPs and patients are established.

Moreover, since affordances for learning were found both within and outside the workplace, workplaces may benefit from offering greater opportunities for collegial interaction, as well as allowing more time for dedicated reading and participating in conferences and courses. How workplaces can facilitate communication with colleagues in other contexts (e.g., during conferences) remains a challenge, given that time allotted for continuous professional development is usually time taken away from clinical duties. Based on these findings, we agree with Hilkenmeier et al. (2021) that there is a need to set aside time for workplace learning in a similar way as applying for time for formal learning activities with participation in courses and conferences. Moreover, it may be appropriate to develop tools and practices that enable GPs to reflect on the triggers for learning they encounter.

### Individual Engagement

According to Billett, workplaces afford opportunities for learning, but the degree of learning is determined by how individuals choose to participate and interact (Billett, 2001; Riera Claret et al., 2020; Tynjälä, 2013). The findings of this study suggest that learning is facilitated by a trustful and safe environment, which has been confirmed in previous studies of GPs (Pype et al., 2014), other medical specialists (Mertens et al., 2018), and nurses (Mlambo et al., 2021). Responsibility for lifelong learning thus lies with both the workplace and the individual clinician (Billett, 2001). Engagement in affordances for learning was linked to individuals’ preferences and personal history of learning, for example by using different web-based sources, or discussing with colleagues. It was also linked to values, norms and procedures around learning, so that while some GPs happily shared questions with local peers, others preferred to turn to external experts (i.e., other GPs and those in other specialties). But even if workplaces provide space for learning by organising clinical meetings, GPs then have to take those opportunities to learn and this is linked to individual agency. In this study, some GPs intentionally (and voluntarily) tried to create an open and safe workplace climate to enhance others’ learning. One interpretation of this is that they were aware of their key role in creating a learning environment, and facilitating others’ learning. However, as is the case in the context of higher education (Elmberger et al., 2019; Strand et al., 2015), such engagement in the learning of peers is rarely rewarded, and hence is reliant on ‘champions’ rather than guided by a clear ambition by the managing organisation. Hence, for health care organisations to facilitate individuals’ engagement in their own learning, and the learning of peers and colleagues, it may require incentivizing such learning (e.g., by setting aside time for peer learning, and to make it explicit and possibly rewarded as part of criteria for promotion). A similar approach has been taken in the area of development of teaching competence in some universities, and has been found successful (Pleschová et al., 2021).

In this study, workplaces offered GPs opportunities to take on different roles that supported lifelong learning, such as acting as supervisors or giving lectures. Taking on such roles, which could be described as intentional guiding learning strategies (Billett, 2001), had reciprocally positive outcomes; not only did it mean that GPs could facilitate others’ learning, but they promoted their own lifelong learning at the same time. Another aspect contributing to a holistic framework for lifelong learning thus includes a purposeful use of such roles.

## Conclusion

In sum, patients, and especially specific patient cases, functions as triggers for GP’s learning, and they engage in different affordances for learning. Further, their engagement is influenced by various workplace aspects. The findings stresses that engagement in affordances for learning is highly intertwined with aspects of the workplace such as organisation, opportunities to take on different roles, climate, and having the space and time to talk. However, learning was also influenced by implicit and explicit policies in the workplace (e.g., when and where to discuss patient cases), formal laws at the national level (e.g., about patient confidentiality), and recommendations on treatments and procedures. An important contribution from this study is hence that healthcare organizations need to take a holistic perspective on physician learning that enables time and engagement in different learning activities as triggered by patients to facilitate lifelong learning.

### Implications for Practice and Future Research

This study highlights the importance of learning between colleagues, which also needs facilitation in the workplace. For this to happen, however, managers and leaders at different levels of health care organisations need to take a holistic approach involving:

- Opportunities for getting to know patients over time.
- Acknowledgements of affordances for learning with colleagues in- and outside the organisation.
- Attention paid to patient workflow, financial incentives to make engagement in the learning of peers rewarded.
- Purposeful use of supervisory roles.
- Establishment of arenas for collegial interaction.

In terms of future research, we recommend to explore how these findings apply to other medical specialties, and other health professions. Also, observational studies regarding medical specialists’ learning could further widen our knowledge on this phenomenon.

### Limitations and Strengths

A limitation of this study was that no observations could be made that could have deepened knowledge about affordances of learning related to tacit knowledge and modelling learning. The same is true of how the workplace’s social environment influences lifelong learning and uneven opportunities for engaging in collegial encounters. The interviews may not have exposed all aspects of tacit knowledge, but they helped GPs reflect on their own lifelong learning, which is something many of them had never done. Further, GP’s practice may differ between countries, with GPs in Sweden rarely working in their own clinic, while this is prevalent elsewhere.

Billett (Billett, 2001) describes individuals’ uneven opportunities to participate in social encounters that afford learning according to factors such as being a ‘newcomer’ or ‘old-timer’, or having different roles and standings. This was, however, not prominent in this study, which may be explained by the fact that only GPs were interviewed. Further, the GPs were in general aware of their learning from colleagues, as well as from trainees and other health care professionals.

## Data Availability

Data not available due to ethical considerations.

## Appendix: Interview guide (translated from Swedish)

Professional background:

- Tell me about your work and your professional background
- Can you remember any situations that have formed your career? Your learning and your continuous work as a GP?

Workplace learning:

- What do you do if you experience a situation at work where you feel a lack of knowledge?
- In what situations at work do you experience that you learn something?
- Did you learn anything today? This week? In what context?
- Can you provide an example of when you learned something meaningful at work? How did it happen?
- What factors at work contribute to your learning?
- Are there any obstacles that hinder your learning at work? What are those?
- Do you ever learn in connection to patients? (exemplify)
- Do you learn from colleagues? In what situation and when?
- What other aspects contribute to your learning at work?

Participation in external formal activities:

- What are your opportunities for participating in CPD activities at work?
- Can you tell me about your participation in CPD activities? Why? Why not?
- How do you benefit from colleagues’ participation in CPD activities?

Knowledge transfer:

- How do you and your colleagues share knowledge at work?
- Can you remember a situation when you or a colleague changed their work due to somebody sharing knowledge?
- Are there any obstacles for sharing knowledge in your work context? If yes, what?

## Declarations

Ethical approval was applied for by The Swedish Ethical Review Authority confirmed that no ethical approval was required for this study, which was performed in accordance with the ethical standards laid down in the 1964 Declaration of Helsinki and its later amendments. Informed consent was received. Dnr 2020–06669.
